# Simultaneous removal of caesium and strontium using different removal mechanisms of probiotic bacteria

**DOI:** 10.1038/s41598-024-57678-8

**Published:** 2024-04-01

**Authors:** Rin Endo, Satoshi Karasawa, Hideki Aoyagi

**Affiliations:** 1 Division of Life Science and Bioengineering, Graduate School of Life and Environmental Sciences, University of Tsukuba, 1-1-1 Tennodai, Tsukuba, Ibaraki 305-8572 Japan; 2 Institute of Life and Environmental Sciences, University of Tsukuba, 1-1-1 Tennodai, Tsukuba, Ibaraki 305-8572 Japan

**Keywords:** Biological techniques, Biotechnology, Microbiology

## Abstract

When radioactive materials are released into the environment due to nuclear power plant accidents, they may enter into the body, and exposing it to internal radiation for long periods of time. Although several agents have been developed that help excrete radioactive elements from the digestive tract, only one type of radioactive element can be removed using a single agent. Therefore, we considered the simultaneous removal of caesium (Cs) and strontium (Sr) by utilising the multiple metal removal mechanisms of probiotic bacteria. In this study, the Cs and Sr removal capacities of lactobacilli and bifidobacteria were investigated. Observation using an electron probe micro analyser suggested that Cs was accumulated within the bacterial cells. Since Sr was removed non metabolically, it is likely that it was removed by a mechanism different from that of Cs. The amount of Cs and Sr that the cells could simultaneously retain decreased when compared to that for each element alone, but some strains showed only a slight reduction in removal. For example, *Bifidobacterium adolescentis* JCM1275 could simultaneously retain 55.7 mg-Cs/g-dry cell and 8.1 mg-Sr/g-dry cell. These results demonstrated the potentials of utilizing complex biological system in simultaneous removal of multiple metal species.

## Introduction

The Chernobyl and Fukushima nuclear power plant accidents released large amounts of radioactive elements into the environment, which remained there for a long time^1^. Caesium (Cs) and strontium (Sr) have a high affinity for living organisms; therefore, when absorbed by the body, they cause prolonged internal exposure^2,3^. Since Cs and Sr are elements in the same group as potassium (K) and calcium (Ca), respectively, they behave similarly to these elements in the body, increasing the probability of cancer in the surrounding tissues^4^. Thus, a method for preventing these metals from being absorbed from the digestive tract is required.

Many of the methods used for environmental purification of metals^5^ cannot be used for removal of metals from the gastrointestinal tract. However, it may be possible to remove them using adsorbents by selecting appropriate edible materials. The ingestion of Prussian blue (iron ferrocyanide (III)) has been proposed as a means of facilitating Cs excretion^4,6,7^. This agent has high Cs-scavenging ability and low toxicity^8^. However, it also has several disadvantages, such as causing abdominal discomfort when ingested and requiring a high cost and large amount of time for collection after excretion^9^. In addition, despite the combined presence of Cs and Sr in many cases, Prussian blue can only remove Cs. Besides, alginic acid is known as an agent used to eliminate Sr from the body^10^. This reagent is found in edible seaweed and can be used without any major disadvantages. Eun et al^9^. fabricated a Prussian blue immobilized alginate aerogel and demonstrated it to be an effective method for removing Cs and Sr simultaneously from seawater. However, to the best of our knowledge, no method has been proposed for removing Cs and Sr simultaneously from the gastrointestinal tract.

In terms of edible removers, it is worth considering microorganisms. Many microorganisms that have been reported to have metal-removal capacities, such as lactobacilli^11–17^ and *Saccharomyces cerevisiae*^18^, have long been used as food products and can be easily consumed. Several mechanisms for metal removal by microorganisms have been reported^19,20^. In a previous study, we demonstrated that *Lacticaseibacillus casei* JCM1134 could adsorb metals, such as Sr, mainly through capsular polysaccharides present on the cell surface^21^. Therefore, if bacteria that adsorb Sr with their cell surface components can remove Cs by another mechanism such as bioaccumulation, it is possible to remove Cs and Sr simultaneously. In this study, we focused on lactobacilli, and bifidobacteria and investigated their potential to remove Cs and Sr.

## Results and discussion

### Distribution of Cs

**Figure 1:**
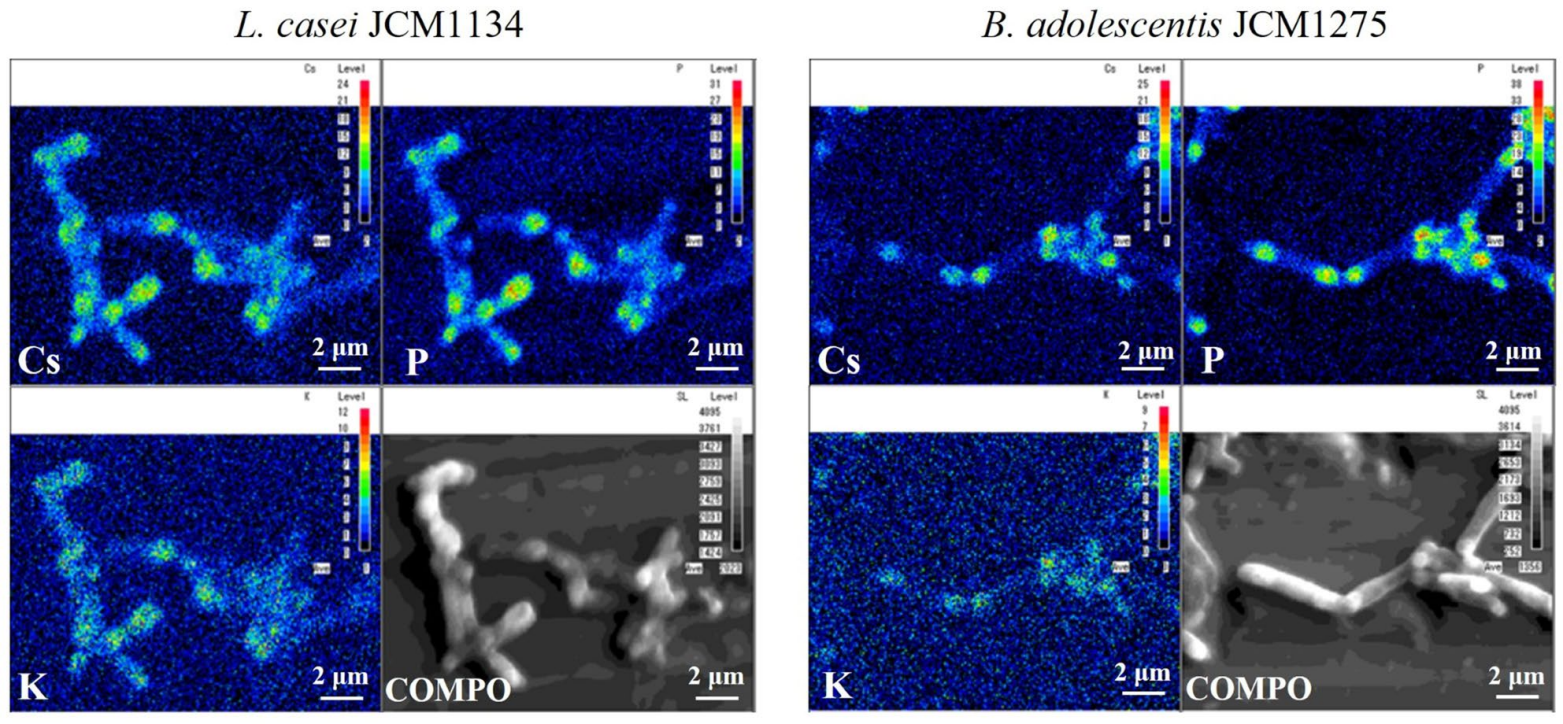
Caesium (Cs), phosphorus (P), and potassium (K) distribution and backscattered electron images (COMPO) of bacterial cells. Element maps show that the concentration of each element is higher at the point where the measurement level is higher.

The elemental distribution of *L. casei* JCM1134 and *Bifidobacterium adolescentis* JCM1275 cultured for 24 h in a medium supplemented with 100 mM Cs was analysed using an electron probe micro analyser. Cs was distributed in a granular form in both strains (Fig. 1). Moreover, phosphorus (P) and K were distributed in high amounts at sites with a high Cs content.

Metal removal by microorganisms can be broadly classified into two categories: bioaccumulation, in which metals are taken up by living bacteria, and biosorption, in which metals are incorporated independently of microbial metabolism, such as adsorption to the surface layer of bacteria^22^. When Cs is removed by biosorption, it should be present evenly in the bacterial body; however, in Fig. 1, Cs was observed to be localized in both bacteria. On the other hand, Cs localization can be explained by considering that it is removed due to bioaccumulation. Some microorganisms store granular substances called polyphosphate inside their cells, which has a negative charge and can trap cations^23^. Therefore, considering that the cations K^+^ and Cs^+^ were trapped by polyphosphate, the results in Fig. 1 that P, K, and Cs were localized in similar locations are consistent. Kuwahara et al^24^. also mentioned the possibility that Cs could be trapped by polyphosphate in their study investigating Cs accumulation in *Streptomyces* sp. K202.

Hence, in *L. casei* JCM1134 and *B. adolescentis* JCM1275, Cs is likely removed via bioaccumulation. This suggests the possibility of simultaneously removing the two representative elements, Cs and Sr, using different removal mechanisms in one microorganism strain.

### Growth inhibition by Cs

**Figure 2:**
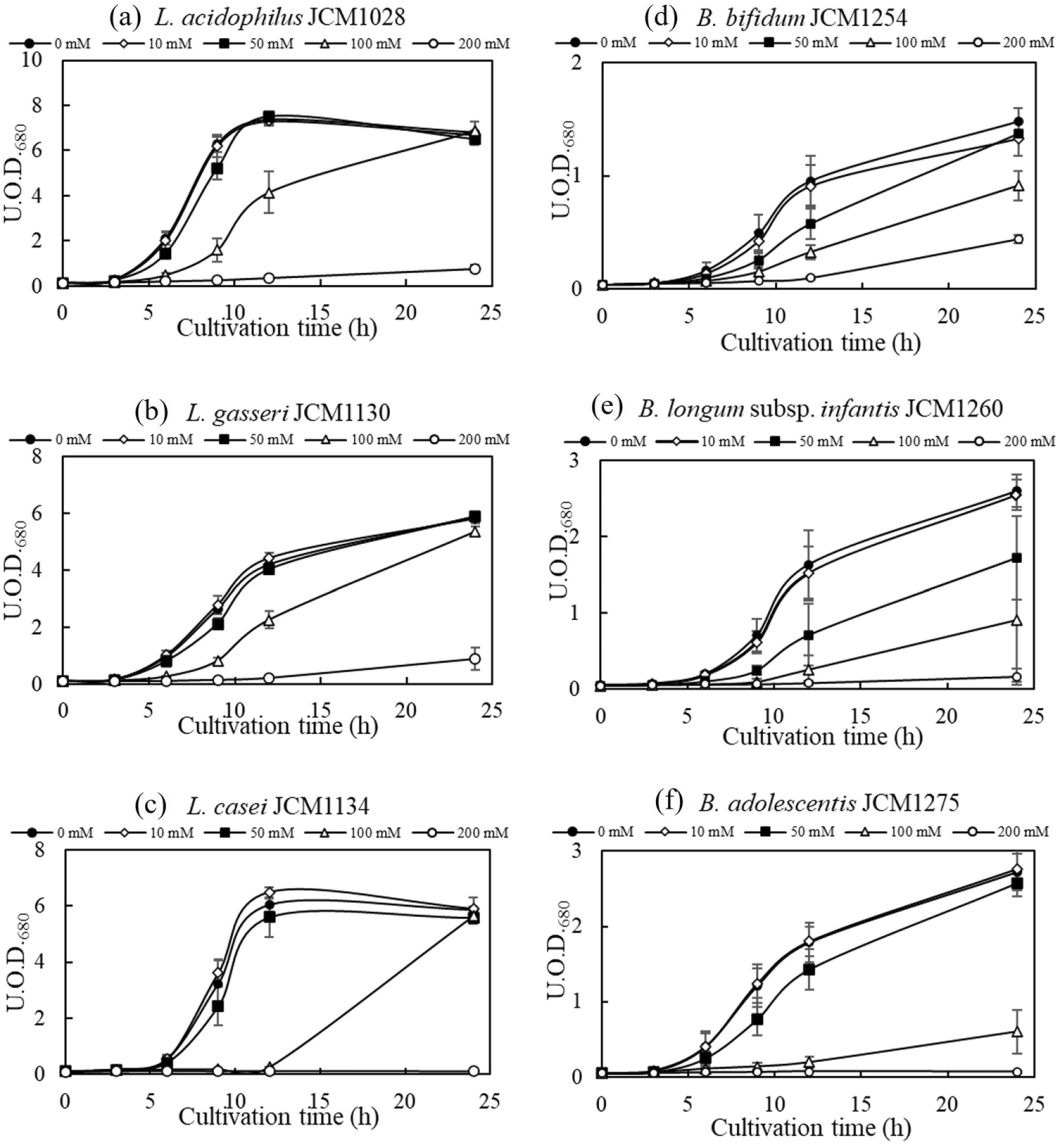
Growth curves of lactobacilli and bifidobacteria on Cs-supplemented medium. The vertical axis shows unit optical density at 680 nm (U.O.D._680_). Each legend indicates the concentration of caesium supplemented to the medium. Error bars represent the standard error (n = 3).

As the growth of microorganisms is inhibited when the Cs concentration is too high^25,26^, we compared the growth of microorganisms to determine the optimal amount of Cs that could support the growth of each microbe (Fig. 2). A Cs concentration of 50 mM had almost no effect on the growth of the lactobacilli strains (Fig. 2a–c); however, at 100 mM or higher, the growth rate and final cell concentration decreased. Three strains of *Bifidobacterium* (Fig. 2d–f) were more susceptible to Cs toxicity than were lactobacilli. For *Bifidobacterium bifidum* JCM1254 (Fig. 2d) and *B. adolescentis* JCM1275 (Fig. 2f), the cell concentrations after 24 h were generally the same at Cs concentrations of 50 mM or less. In contrast, in *Bifidobacterium longum* subsp. *infantis* JCM1260 (Fig. 2e), both the growth rate and final cell concentration decreased at 50 mM. Based on these results, Cs concentration of 50 mM was used for later experiments to minimize growth inhibition by Cs.

It should be noted that a Cs concentration of 50 mM is not realistic, considering the actual gastrointestinal environment. For example, the concentration of ^137^Cs in food collected after the Chernobyl nuclear accident was as high as 1 × 10^4^ Bq/kg^1^. When this was converted to a molar concentration assuming that 1 kg represents 1 L, it was approximately 2 × 10^−11^ M, which was far lower than the Cs concentration used in the present study. However, the K concentration has a large effect on the amount of Cs removed because they behave similarly^27–29^. The concentrations of K and other substances in the culture environment did not indicate an environment suitable for the gastrointestinal tract. We considered that there was no reason to set only the Cs concentration at a realistic value. Hence, an evaluation under practical conditions was not conducted in the present study, and the possibility of simultaneous removal of Cs and Sr was explored.

### Cs removal capacity

**Figure 3:**
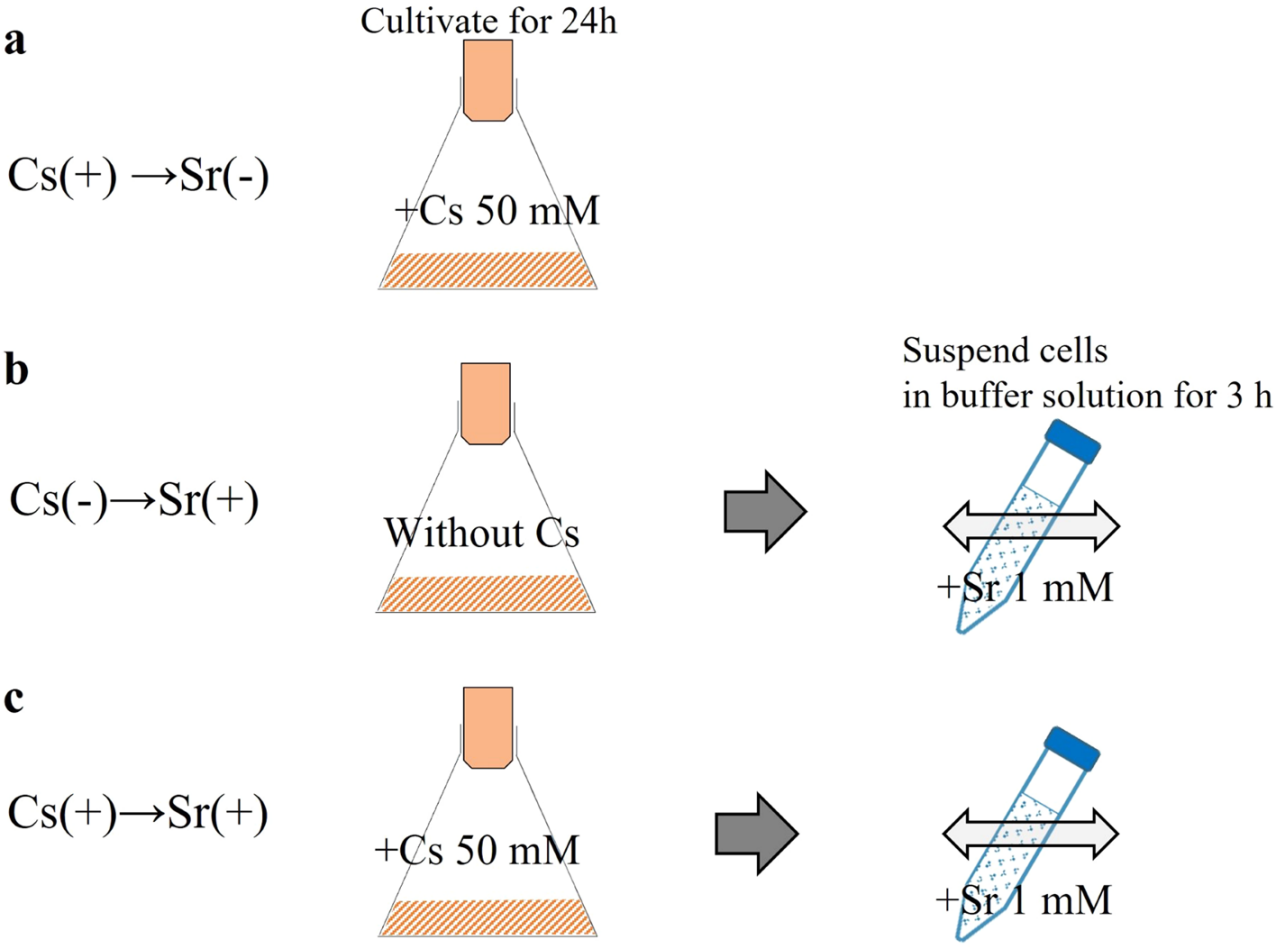
Experimental design for removal of Cs and Sr by bacteria.

**Figure 4:**
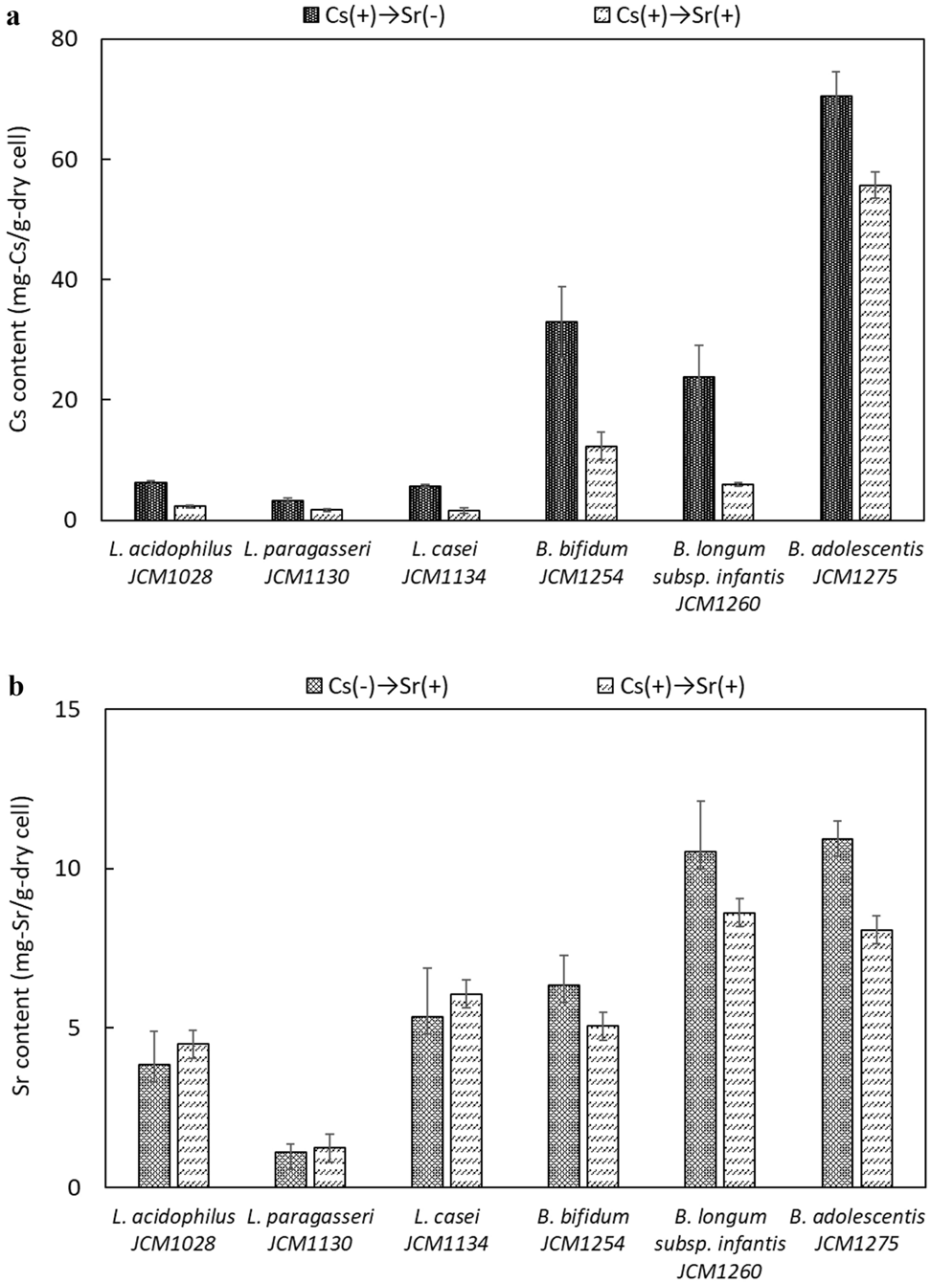
Amount of Cs and Sr contained in each bacterium. Cs ( +) indicates the addition of 50 mM Cs to the culture medium, and Sr (+ ) indicates the suspension of cells in the solution containing 1 mM Sr. Error bars represent the standard error (n = 3).

To evaluate the amount of Cs that could be removed when only Cs was present (Fig. 3a), the Cs content of each strain was measured. As a result, 3.3–6.3 mg-Cs/g-dry cells were detected in lactobacilli and 23.8–70.5 mg-Cs/g-dry cells in *Bifidobacterium* (Fig. 4a). The accumulation of Cs was confirmed in all the bacteria used, and large differences were observed in the Cs accumulation capacity depending on the species.

We compared our current results on the Cs removal capacity with those previously reported for other Cs-removing bacteria. Kato et al^30^. investigated the Cs content per unit number of bacteria by Cs-resistant bacteria and reported 142.2 ± 7.1 mg-Cs/g-dry cell and 61.5 ± 6.9 mg-Cs/g-dry cell for *Streptomyces* sp. TOHO-2 and *Streptomyces lividans* TK24, respectively. Tomioka et al*.* reported 92 mg-Cs/g-dry cells for *Rhodococcus erythropolis* CS98 and 52 mg-Cs/g-dry cells for *Rhodococcus* sp. strain CS402^26^. The results of the present study showed that the three strains of lactobacilli had lower Cs content than the *Bifidobacterium* strains, with the highest removal capacity observed for *B. adolescentis* JCM1275 (70.5 mg-Cs/g-dry cells). The Cs removal capacity of *B. adolescentis* JCM1275 in the present study was similar to that previously reported for other Cs-removing bacteria.

### Sr removal capacity

The Sr removal capacity was evaluated by suspending the cells cultured for 24 h in a buffer containing Sr and then measuring the amount of Sr contained in the cells (Fig. 3b). Sr was detected in the cells of all the strains used: 1.1–5.3 mg-Sr/g-dry cell for lactobacilli and 6.3–10.9 mg-Sr/g-dry cell for *Bifidobacterium* (Fig. 4b).

Based on our results, it is plausible that Sr is removed by biosorption, which occurs independently of bacterial metabolism, as Sr removal occurred without a nutrient source. In previous reports, the mechanism of Sr removal using microorganisms was considered to be through biosorption occurring on the surface of bacteria^13,31^. In addition, our previous study suggested that *L. casei* JCM1134 removed Sr via adsorption^21^.

Comparing the amount of Sr removed in the present study with that of other previously reported microbial adsorbents, 24.4 mg-Sr/g-dry cell was reported for *Rhizopus nigricans*^32^, 6.6 mg-Sr/g-dry cell for *Rhizopus arrhizus*^33^, and 10.0 mg-Sr/g-dry cell for *S. cerevisiae*^18^. Although making a direct comparison is difficult because the removal ability of microorganisms varies with culture and adsorption conditions, the Sr removal capacity of *Bifidobacterium* (6.3–10.9 mg-Sr/g-dry cell) in our study was similar to those previously reported for other microbial adsorbents. On the other hand, the removal ability of lactobacilli (1.1–5.3 mg-Sr/g-dry cell) was slightly lower than that of the previously reported microorganism adsorbents.

### Simultaneous removal of Cs and Sr

To examine the amounts of Cs and Sr that one type of microbial strain can simultaneously retain, bacterial cells cultured in Cs-supplemented medium were suspended in Sr-containing buffer (Fig. 3c). As mentioned above, we expected the amounts of Cs and Sr to be irrelevant because they were thought to be removed by different mechanisms. However, contrary to expectations, all the strains showed a decrease in Cs content after Sr adsorption. The amount of released Cs differed depending on the strain, with *B. adolescentis* JCM1275 and *B. longum* subsp. *infantis* JCM1260 releasing 21% and 75%, respectively (Fig. 4a). In contrast, the amount of Sr removed was almost the same between cells cultured in the normal medium and those cultured in the Cs-supplemented medium (Fig. 4b).

The release of Cs after Sr adsorption can be explained by considering multiple mechanisms for Cs removal. As shown in Fig. 1, Cs is most likely retained in bacteria by trapping in polyphosphate; however, this does not mean that other mechanisms are not involved. Some previously published studies have reported Cs biosorption by the surface components of bacteria^13,28,34,35^, and it is possible that the microorganisms used in this study also adsorbed Cs on the surface. Therefore, the adsorbed Cs^+^ may have been replaced by the highly charged Sr^2+^ and released.

Although a certain amount of Cs was released, *B. adolescentis* JCM1275 showed high removal capacities of 55.7 mg-Cs/g-dry cell and 8.1 mg-Sr/g-dry cell. This indicates that with appropriate strain selection, it may be possible to remove multiple radioactive materials using a single type of microorganism.

Because some microbial functions change depending on the environment, it is necessary to investigate whether similar effects can be expected in an in vivo environment. Through these studies, it is expected that probiotic bacteria can be used as a highly accessible method for excreting radioactive elements from the gastrointestinal tract through fecal excretion.

### Conclusion

Cs has been suggested to bioaccumulate in lactobacilli and *Bifidobacterium*. Therefore, we investigated whether Cs and Sr can be removed simultaneously using two mechanisms, bioaccumulation and biosorption, in combination with the metal-adsorption function of microorganisms. We showed that *B. adolescentis* JCM1275 simultaneously retained these two metals at high concentrations. If these functions were replicated in the gastrointestinal tract, they could be used to inhibit the absorption of multiple radionuclides into the human body.

## Methods

### Reagents

Glucose, K_2_HPO_4_, Polysorbate 80 (Tween 80; for biochemistry grade), sodium ascorbate (Wako special grade), l-cysteine hydrochloride, CsCl, H_2_O_2_, and SrCl_2_, were obtained from Fujifilm Wako Pure Chemical Co. (Osaka, Japan). These reagents without grade description were of guaranteed grade. Casein peptone and yeast extract were from Nihon Pharmaceutical Co. Ltd. (Osaka, Japan). MRS medium and beef extract were from Becton, Dickinson and Co. (Franklin Lakes, NJ, USA). Bis–Tris was obtained from MP Biomedicals (Santa Ana, CA, USA).

### Culture conditions

Lactobacilli (*Lactobacillus acidophilus* JCM1028, *Lactobacillus paragasseri* JCM1130, and* L. casei* JCM1134) and bifidobacteria (*B. bifidum* JCM1254, *B. longum* subsp. *infantis* JCM1260, and* B. adolescentis* JCM1275) were provided by the Japan Collection of Microorganisms, RIKEN BRC which is participating in the National BioResource Project of the MEXT, Japan. Lactobacilli were cultured in MRS medium. The bifidobacterial medium contained 10 g/L casein peptone, 5.0 g/L beef extract, 5.0 g/L yeast extract, 10 g/L glucose, 3.0 g/L K_2_HPO_4_, and 1.0 mL/L Tween 80; 1.0% (w/v) sodium ascorbate and 0.05% (w/v) l-cysteine hydrochloride were added after autoclaving. Both bacteria were statically incubated at 37 °C. All experiments were performed after two pre-cultures with 1% (v/v) inoculum.

### Microbial growth on Cs medium

CsCl was added to the medium at concentrations of 0, 10, 50, 100, and 200 mM, and each bacterium was cultured statically at 37 °C. Non-radioactive Cs was used in this study. The concentration of bacteria in the culture medium was monitored by measuring absorbance at 680 nm using a spectrometer UV-1200 (Shimadzu Co., Kyoto, Japan).

### Element mapping

Bacteria cultured for 24 h in Cs-supplemented medium were washed with distilled water and diluted 20 times for lactobacilli and 10 times for *Bifidobacterium*. Thereafter, the bacterial culture solution was dropped onto a sample table, dried, and carbon deposition was performed using a vacuum deposition apparatus JFE-420 (JEOL Ltd., Tokyo, Japan). Backscattered electron and elemental mapping images of Cs, P, and K were obtained using an electron probe micro analyser JXA-8530F (JEOL Ltd.) at an accelerating voltage of 10.0 kV and a beam current of 5.0 × 10^−8^ A.

### Determination of Cs contained in bacterial cells

The culture medium was incubated for 24 h with Cs concentration of 50 mM and centrifuged (6000 × *g*, 3 min) to collect the bacterial pellets, which were then suspended in distilled water and centrifuged twice to remove components derived from the culture medium. Wet digestion of bacterial cells was performed using a method modified from Tomioka et al^26^. The bacterial pellets were transferred to a conical beaker, 5 mL of nitric acid was added, and the watch glass was placed on the conical beaker and heated at 180 °C for 2–3 h. To decompose the sample, 0.5 mL of H_2_O_2_ was added until the colour of the solution became light pale yellow to colourless. The Cs concentration was determined using inductively coupled mass spectrometry (ICP-MS; ELAN DRC-e; PerkinElmer, Waltham, MA, USA) after dilution to the detectable concentration. The Cs content was converted to concentration per unit dry bacterial mass (mg-Cs/g-dry cell).

### Sr adsorption amount

The experiment was conducted using a method modified from our previous study^21^. In brief, 2 mL of the culture medium was incubated for 24 h as described in section “Culture conditions” or “Determination of Cs contained in bacterial cells” was sampled and the bacteria were collected by centrifugation (6000 × *g*, 3 min). After washing, the bacteria were suspended in 2 mL of Bis–Tris buffer (1 mM, pH 6.8), SrCl_2_ (non-radioactive) was added to a final Sr concentration of 1 mM, and the bacterial suspension was incubated at 37 °C for 3 h (200 strokes/min, stroke length = 25 mm). After incubation, the bacterial cells were collected by centrifugation (6000 × *g*, 3 min), washed well, and subjected to acid decomposition as described in the “Determination of Cs contained in bacterial cells” section. The Sr content was then determined by ICP-MS.

## Data Availability

All data presented in this study are available from the corresponding author upon reasonable request.
